# Intracranial compliance monitoring using pulse shape index in traumatic brain injury: relation to cerebral physiology and clinical outcome

**DOI:** 10.1186/s13054-026-06071-0

**Published:** 2026-05-09

**Authors:** Teodor Svedung Wettervik, Erta Beqiri, Anders Hånell, Agnieszka Kazimierska, Magdalena Kasprowicz, Peter Smielewski

**Affiliations:** 1 Department of Medical Sciences, Section of Neurosurgery, Uppsala University, Uppsala, SE-751 85 Sweden; 2 Brain Physics Laboratory, Department of Clinical Neurosciences, Division of Neurosurgery, University of Cambridge, Cambridge, UK; 3 Department of Biomedical Engineering, Faculty of Fundamental Problems of Technology, Wroclaw University of Science and Technology, Wroclaw, Poland

**Keywords:** Intracranial compliance, Intracranial pressure, Neurocritical care, Pulse shape index, Traumatic brain injury

## Abstract

**Background:**

The intracranial pressure (ICP) pulse waveform reflects intracranial compliance. The pulse shape index (PSI), an artificial intelligence (AI)-based metric ranging from 1 (normal) to 4 (disturbed), quantifies morphological waveform pathologies. Early findings in smaller cohorts indicate that elevated PSI is associated with mass lesions, aging, higher ICP, and worse outcome. This study examined how PSI relates to other markers of intracranial compliance, the risk of ICP crisis, and clinical outcome in a large traumatic brain injury (TBI) cohort.

**Methods:**

This retrospective study included 321 TBI patients with ≥ 12 h of ICP/PSI monitoring. PSI was analysed in relation to ICP, ICP pulse amplitude (AmpICP), the moving correlation coefficient between mean ICP and ICP amplitude (RAP index), and arterial blood pressure (ABP) using generalised additive models (GAMs). Linear mixed-effects models assessed whether PSI during preceding hours predicted later ICP elevations (e.g., ICP > 20/22 mmHg). The prognostic value of PSI was tested using univariable Mann–Whitney U test and multivariable (after adjustment for age, Glasgow Coma Scale, ICP, CPP, and PRx) logistic regression for mortality (Glasgow Outcome Scale = 1) and favourable outcome (Glasgow Outcome Scale = 4–5).

**Results:**

PSI increased with higher ICP, AmpICP, RAP, and ABP. PSI during the preceding hour predicted increased ICP burden above 20 mmHg (marginal R^2^ ~30%). PSI was lower in survivors and in patients with favourable outcomes. However, in multivariable logistic regressions, PSI was not independently associated with mortality or favourable outcome.

**Conclusions:**

PSI was linked to established indicators of intracranial compliance and provided early warning of impending intracranial hypertension. While PSI showed univariable associations with outcome, these did not remain in multivariable models. Retrospectively, PSI does not appear to have independent prognostic value, but rather a complementary value. Prospectively, it may serve as a dynamic marker of deteriorating intracranial compliance and an early signal for future ICP crisis.

**Supplementary Information:**

The online version contains supplementary material available at 10.1186/s13054-026-06071-0.

## Introduction

Elevated intracranial pressure (ICP) is strongly associated with unfavourable outcomes in traumatic brain injury (TBI) [1–3], and neurocritical care (NCC) management aims to maintain ICP below 22 mmHg [2]. Intracranial compliance indicates to what extent additional intracranial volume, e.g., from haemorrhage and oedema, will increase ICP [4–6]. Such information is of great importance for predicting future ICP crises [7] and evaluating the effectiveness of NCC interventions [8, 9].

Historically, intracranial compliance in the NCC setting was assessed by inflating an intracranial balloon and observing the resulting rise in ICP [10, 11], but safety issues led to its clinical abandonment [10, 11]. Currently, less invasive approaches are used, primarily deriving information from the ICP waveform [5, 10]. One widely used parameter is ICP pulse amplitude (AmpICP), reflecting the height of the ICP pulse wave generated by cardiac stroke volume [12, 13]. AmpICP increases as the intracranial pressure–volume curve steepens and has been associated with worse primary brain injury [8], raised ICP [14], and unfavourable outcome [15] in TBI. The compensatory reserve index, RAP, a moving Pearson correlation coefficient between 10-second values of mean ICP and AmpICP over 5 min, further characterises intracranial compliance [4, 5, 10]. RAP approaches zero during the flat portion of the intracranial pressure–volume curve, increases towards one in the steep phase, and turns negative at critically high ICP levels due to cerebrovascular collapse [4]. However, both AmpICP and RAP have limitations: AmpICP may be influenced by factors not necessarily related to intracranial compliance such as arterial blood pressure (ABP) [10, 16], while RAP is susceptible to baseline effects errors in mean ICP due to spontaneous fluctuations and sensor malfunction, and should generally be interpreted as a qualitative rather than quantitative index of cerebral compliance [17].

Beyond pulse amplitude-derived measures, it is well known that the morphology of the ICP pulse waveform changes with impaired compliance, but this transition has been challenging to quantify. Under normal intracranial conditions, the systolic peak (P1) of the ICP waveform exceeds the tidal (P2) and dicrotic (P3) peaks. As intracranial compliance decreases, P2 and P3 surpass P1, and the dicrotic notch ultimately becomes indistinct. Based on these transitions, it has been suggested that ICP waveforms can be classified into four morphological types, ranging from normal (class 1) to severely pathological (class 4) [14, 18–20]. Recently, the pulse shape index (PSI) has been suggested as an artificial intelligence (AI)-based continuous metric to quantify ICP pulse waveform pathologies using these four classes, and adding one class for artefactual waveforms [10, 17, 19, 20]. Initial studies have shown promising results, with higher PSI values strongly associated with radiological signs of mass effect [14, 19], higher ICP [7, 14, 15], aging [15] and worse outcomes [14, 19, 20]. In addition, PSI was shown to be one of the factors that explained AI-based predictions of ICP crises [7]. However, as a novel parameter, PSI remains in an early phase of validation, and its accuracy and generalisability across different patient populations have yet to be firmly established. Moreover, it remains uncertain whether PSI carries independent prognostic value after adjustment for ICP and other established NCC variables.

Thus, there is a need to further investigate the potential and limitations of this novel metric of intracranial compliance. In this study, the first aim was to examine PSI in relation to ICP and established measures of intracranial compliance. The second was to evaluate the ability of PSI to predict ICP crises without the AI framework. The third was to assess the association between PSI and clinical outcome. We hypothesised that PSI would be moderately related to other measures of ICP dynamics, as it reflects overlapping but partly distinct aspects of intracranial physiology; that it would reasonably predict later ICP crises; and that although higher PSI would be associated with worse outcome, traditional predictors, including age and ICP, would remain the main determinants of outcome in multivariable analyses.

## Materials and methods

### Patients and study design

In this observational, retrospective, single-centre study, neuromonitoring and demographic data from 781 TBI patients admitted in the NCC unit of the Addenbrooke’s Hospital (Cambridge, UK) between 2002 and 2022 were accessed from the Brain Physics research database [21]. Two hundred five patients without available outcome data, 213 treated with decompressive craniectomy (DC) or with no data on DC surgery, and 42 with less than 12 h of ICP/PSI data were excluded. Thus, 321 patients were included in the final dataset.

### Management

The management protocol has been described in detail in previous studies [22, 23]. ICP- and partial brain tissue oxygen tension (pbtO_2_)-monitoring were considered in unconscious TBI patients. Overall, ICP was targeted below 20 or 25 mmHg (the latter threshold for barbiturates and DC), cerebral perfusion pressure (CPP) above 60 mmHg, pbtO_2_ above 15 to 20 mmHg, partial pressure of carbon dioxide within 4.5–5 kPa, and arterial glucose within 6 to 8 mmol/L. The pbtO_2_ target was mainly managed by ICP-lowering and CPP-augmenting measures. Pressure reactivity index (PRx) and optimal cerebral perfusion pressure (CPPopt), available at the bedside since 1999 and 2012, respectively, were not actively targeted, but could be taken into account based on the discretion of the treating physician.

### Clinical data and outcome

Baseline characteristics and demographic data retrieved from the database were: age, sex, Glasgow Coma Scale (GCS) at admission, and pupillary reactivity.

Clinical outcome was assessed as Glasgow Outcome Scale (GOS) score at 6 months post-injury, either as a clinical assessment or via telephone interviews by trained staff. GOS ranges from 1 (death) to 5 (good recovery) [24, 25]. Outcome was dichotomized as favourable vs. unfavourable (GOS 4–5 vs. 1–3) and survival vs. mortality (GOS 2–5 vs. 1).

### Collection and processing of physiological data

ICP was monitored using an intraparenchymal probe (Codman ICP MicroSensor, Codman & Shurtleff, Raynham, Massachusetts). Arterial blood pressure (ABP) was recorded from the radial or femoral artery (Baxter Healthcare, Deerfield, Illinois), with zero calibration at the level of the heart (2002–2015) and the tragus (2015–2022). Physiological signals were continuously streamed to the ICM+ software platform (Cambridge Enterprise, University of Cambridge, UK; t a sampling frequency ranging from 50 to 250 Hz [26].

ICP values below − 30 and above 200 were rejected for all subsequent calculations. All per-patient data processing was performed using ICM+. PSI, which was originally trained on ICP waveform data from Wroclaw [20], and was calculated in the present study using an ICM+ plugin implementing a previously validated deep neural network [10, 14, 19, 27] which classifies each pulse waveform into one of four classes (1 to 4), representing a spectrum from normal to increasingly pathological shapes. Artefactual pulses were automatically labelled by the classification model and subsequently excluded from further analysis. PSI was then calculated in consecutive non-overlapping 5-minute windows as a weighted average of class numbers, with weights representing the occurrence of each class, capturing continuous changes in waveform morphology. The peak-to-peak pulse amplitude was computed as the maximum minus minimum ICP within a 1.5-second window, updated every second. AmpICP was then defined as the median of these min-to-max values calculated over non-overlapping 5-minute windows. AmpICP values were considered valid if they fell between 0.04 and 40 mmHg [28]. ABP and ICP were down-sampled to 0.1 Hz after automatic and manual artefact removal for calculations of mean values, as previously described [29]. RAP was calculated as the moving Pearson correlation of 10-second averages of mean ICP and frequency domain-based pulse amplitude of ICP (calculated as the amplitude of the fundamental harmonic of ICP) over 5 min, and updated every minute [4]. PRx was calculated as the moving Pearson correlation coefficient of 30 consecutive 10-second average values of ABP and ICP, updated every minute [30, 31]. All the physiological variables were analysed over the entire period of monitoring based on minute-by-minute data, thus both PSI and AmpICP were up-sampled from 0.0033 to 0.017 Hz. Minute-by-minute CPP was calculated as ABP minus ICP. In some specific analyses, described below, the data were analysed as hourly averages. The good monitoring time (GMT) was defined as the remaining coverage of data after artefact removal for each physiological variable. The median value of PSI, AmpICP, RAP, ICP, PRx, and CPP were calculated based on the entire monitoring time. Furthermore, the %GMT of PSI was calculated within each of the six 0.5-intervals from 1 to 4. The choice of 0.5-wide intervals provided a balance between resolution and stability: the intervals were narrow enough to capture clinically meaningful variation in PSI, yet wide enough to avoid excessive fragmentation of the data and retain robust sample sizes within each bin.

### Statistical analysis

Statistical analyses were performed using R version 4.2.3 (2023-03-15 ucrt; R Foundation for Statistical Computing, Vienna, Austria) in RStudio version 2023.09.1 [32]. Categorical variables were summarised as counts and proportions, while continuous and ordinal variables were reported as medians with interquartile ranges (IQR). *First*, associations between PSI and physiological variables related to systemic physiology (ABP) and intracranial compliance (ICP, AmpICP, and RAP) were visualised using generalised additive models (GAMs) with cubic splines and restricted maximum likelihood estimation. For the GAMs of AmpICP and RAP versus PSI, sensitivity analyses were performed excluding data points where ICP simultaneously exceeded 40 mmHg. This threshold was chosen because AmpICP may artifactually decrease at very high ICP levels, potentially distorting the modelled relationships. Such extreme ICP values were rare in the exploratory analysis, but were nonetheless removed to ensure the robustness of the results [4]. For the GAM of AmpICP vs. PSI, we also performed a sensitivity analysis stratified by age (≤ median vs. > median), as PSI may be strongly influenced by this demographic factor [15]. These models were based on minute-by-minute data using the *geom_smooth* function from the *ggplot2* package, enabling population-level exploration of potential non-linear relationships. *Second*, to explore PSI as a predictor of raised ICP metrics (mean/median ICP, %GMT of ICP > 20/22 mmHg [i.e., local and Brain Trauma Foundation guideline threshold, respectively] etc.), we first explored linear mixed-effects models using averaged hourly values of physiological data, with PSI at 1, 2, and 3 h, respectively, earlier as the main predictors and mean ICP at the corresponding lag as a covariate. Models included random intercepts with the patient specified as the random-effects grouping factor. For the most meaningful dependent variables we explored models with time as random slope, allowing the ICP temporal trends to change over time within patients, and with PSI at the correspondent lag as slope, acknowledging that the effect that PSI has on the dependent variable also may change between patients. Model fit was compared across candidate random-effects structures, and residual diagnostics were visually inspected. Given the longitudinal nature of the data, residual autocorrelation was also explored using an AR(1) correlation structure. *Third*, group comparisons of PSI values across outcome categories (mortality vs. survival; favourable vs. unfavourable outcome) were performed using the Mann–Whitney U test. In addition, multivariable logistic regression analyses were conducted to assess the association between PSI and outcome, expressed as odds ratios (OR) with 95% confidence intervals (CI). Two models were built: the first adjusted for demographic (age) and injury severity (GCS) variables; the second additionally adjusted for key NCC targets (ICP, PRx, and CPP). A p-value < 0.05 was considered statistically significant. No adjustment for multiple comparisons was performed.

### Visualizations of the relation between PSI and outcome

The association between PSI and patient outcome was also assessed using two complementary visualisation approaches. In the first approach, based on a single variable [33], a univariable outcome heatmap was generated based on the %GMT spent at different PSI levels. The PSI range (1 to 4) was divided into six discrete grid cells (0.5-unit intervals). For each cell, the %GMT per patient was calculated and correlated with outcome expressed by GOS using Spearman’s rank correlation (r). The resulting correlation coefficients were visualised using the jet colour scale, where blue indicated a favourable association and red an unfavourable one. Given the moderate strength of observed correlations (*r* ≤ 0.3), the colour scale was restricted to this range. To produce smoother images, each pixel was subdivided into a 6 × 6 grid of subpixels, followed by application of a Gaussian kernel filter with a standard deviation of 1 pixel. Grid cells with fewer than five patients contributing at least 5 min of GMT were coloured white. A corresponding density heatmap was constructed by counting the number of observations within each grid cell, normalised per patient by the highest cell count, also using a similar smoothing procedure as mentioned above. In this density heatmap, blue denoted frequent and red rare observations.

In the second approach, based on two variables [34, 35], potential interactions between PSI and key NCC targets, including ICP, PRx, and CPP, were evaluated in relation to outcome expressed by GOS. Two-dimensional outcome heatmaps were created by combining PSI (six grid cells; 0.5-unit intervals) with each respective parameter. For the PSI/ICP analysis, the grid comprised 120 cells (6 × 20), with ICP ranging from 0 to 40 mmHg in 2 mmHg intervals. Similarly, 120 cells (6 × 20) were used in the PSI/PRx plot (PRx range: − 1 to + 1, 0.10-unit intervals), and 180 cells (6 × 30) were used in the PSI/CPP plot (CPP range: 40 to 100 mmHg, 2 mmHg intervals). For each cell in these plots, the %GMT across all patients was computed and correlated with GOS using Spearman’s correlation. Correlation coefficients were visualised using the same jet colour scale (blue = favourable, red = unfavourable), restricted to a correlation coefficient range of ± 0.3. Similar smoothing procedures to that described above was applied. Grid cells with insufficient data (fewer than five patients with at least 5 min of GMT) were coloured white. Corresponding density heatmaps were constructed using the same methodology as in the univariable analysis. Similar smoothing procedures to the single-variable analysis described above was applied.

## Results

### Demography, admission variables, and outcome

In the cohort of 321 TBI patients (Table 1), the median age was 45 (IQR 25–55) years and the male/female ratio was 76/24%. The median GCS was 7 (IQR 4–9) and 18% of the patients exhibited one or two unreactive pupils at admission. At six months post-injury, the median GOS was 4 (IQR 3–4), 56% of the patients had recovered favourably, and 18% were deceased.

**Table 1:** Demography, admission variables, and outcome

Variables	
Patients, n (%)	321 (100%)
Age (years), median (IQR)	45 (25–55)
Sex (female/male), n (%)	71/229 (24/76%)
GCS, median (IQR)	7 (4–9)
Pupillary reactivity (intact/1 unreactive/2 unreactive), n (%)	151/29/5 (82/16/3%)
GOS, median (IQR)	4 (3–4)
Favourable outcome, n (%)	181 (56%)
Mortality, n (%)	57 (18%)

Missing data: Sex (*n* = 21), pupillary reactivity (*n* = 136)

GCS = Glasgow Coma Scale. GOS = Glasgow Outcome Scale. IQR = Interquartile range. n = number of patients

### Cerebral physiology during the monitoring period

Out of the whole monitoring period (median 161 [IQR 77–243] hours), PSI data was available for a median of 98 (IQR 49–164) hours, while ICP data was available for 103 (IQR 54–179) hours. The median proportion of available PSI during ICP monitoring was 97% (IQR 90–100, range 10–100). The median PSI was 1.8 (IQR 1.0–2.7), with the highest %GMT within the 1.0–1.5 range (median 40% [IQR 6–82]). The cerebral physiological variables are described in detail in Table 2.

**Table 2:** Cerebral physiology – descriptive data averaged over the full GMT

Variables	
*NCC targets*	
ICP (mmHg), median (IQR)	14 (10–17)
ABP (mmHg), median (IQR)	91 (85–95)
CPP (mmHg), median (IQR)	76 (72–80)
PRx (a.u.), median (IQR)	+ 0.02 (−0.11 to + 0.15)
*Intracranial compliance measures*	
AmpICP (mmHg), median (IQR)	6.3 (5.1–8.0)
RAP (a.u.), median (IQR)	0.79 (0.64–0.88)
PSI (a.u.), median (IQR)	1.8 (1.0–2.7)
PSI within threshold (%GMT)	
1.0–1.5, median (IQR)	40 (6–82)
1.5–2.0, median (IQR)	9 (3–18)
2.0–2.5, median (IQR)	8 (2–17)
2.5–3.0, median (IQR)	5 (1–17)
3.0–3.5, median (IQR)	4 (0–22)
3.5–4.0, median (IQR)	0 (0–1)

ABP = Arterial blood pressure. AmpICP = peak-to-peak ICP pulse amplitude. a.u. = arbitrary units. CPP = Cerebral perfusion pressure. GMT = Good monitoring time. ICP = Intracranial pressure. IQR = Interquartile range. NCC = Neurocritical care. PRx = Pressure reactivity index. PSI = Pulse shape index. RAP = the correlation (r) between ICP amplitude and ICP

### PSI in relation to systemic physiology and intracranial compliance

**Fig. 1:**
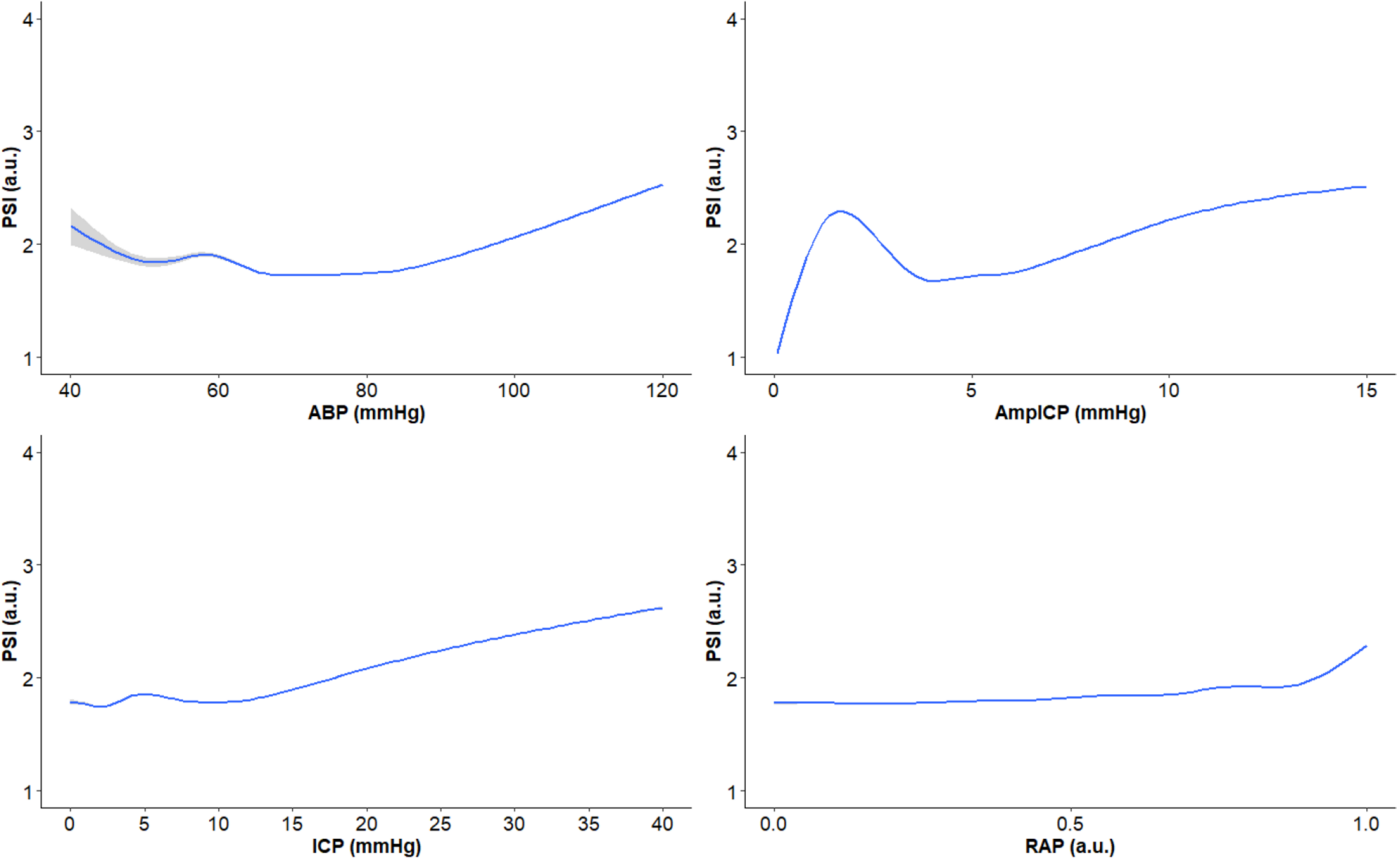
PSI in relation to perfusion and intracranial compliance. These GAMs illustrate the potentially non-linear relationships between PSI and ABP, ICP, AmpICP, and RAP. Each analysis was performed within physiologically relevant ranges: PSI 1–4, ABP 40–120 mmHg, ICP 0–40 mmHg, AmpICP 0–15 mmHg, and RAP 0.0 to + 1.0. AmpICP = peak-to-peak ICP pulse amplitude. ABP = Arterial blood pressure. GAM = Generalised additive models. ICP = Intracranial pressure. RAP = the correlation (r) between ICP amplitude and ICP

**Fig. 2:**
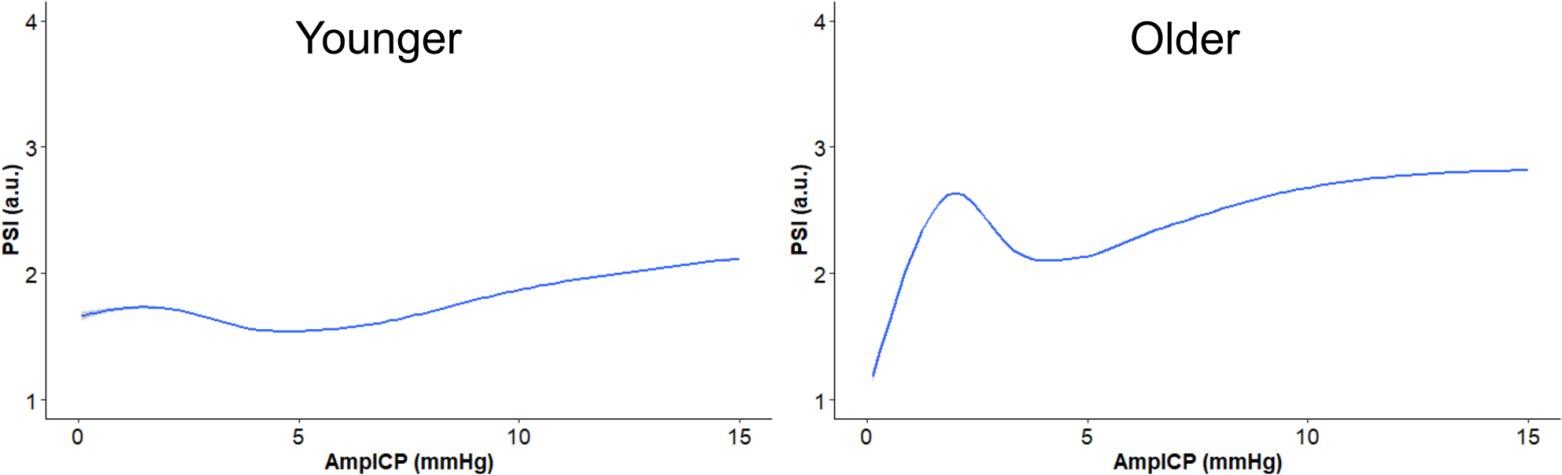
PSI in relation to AmpICP for different age groups. These sensitivity analyses illustrate the potentially non-linear relationships between PSI and AmpICP, modelled using GAMs, for a younger (age ≤ median) and older (age > median) sub-cohort, with the median in this group equal to 45 years. Similar patterns were observed across multiple age dichotomizations, including when applying relatively higher age thresholds. Each analysis was restricted to physiologically relevant ranges: PSI 1–4 and AmpICP 0–15 mmHg. AmpICP = peak-to-peak ICP pulse amplitude. GAM = Generalised additive models. ICP = Intracranial pressure. PSI = Pulse shape index

As illustrated by the GAM analyses in Fig. 1, PSI increased with ABP, particularly at values of 80 mmHg and above. PSI was volatile at low AmpICP (0–5 mmHg), with an early prominent increase followed by a decrease, but subsequently showed a steady increase with higher AmpICP. A slight increase in PSI was observed with higher RAP, especially at values exceeding + 0.5. Excluding data points when ICP was above 40 mmHg did not impact the GAMs of PSI vs. AmpICP and RAP, respectively (Additional File 1). Dichotomising age at the median showed that the volatile PSI pattern at low AmpICP was mostly confined to the older group as compared with younger patients (Fig. 2). PSI remained stable within an ICP range of 0–15 mmHg, but increased more steeply at higher ICP levels.

### PSI as a predictor of ICP

**Fig. 3:**
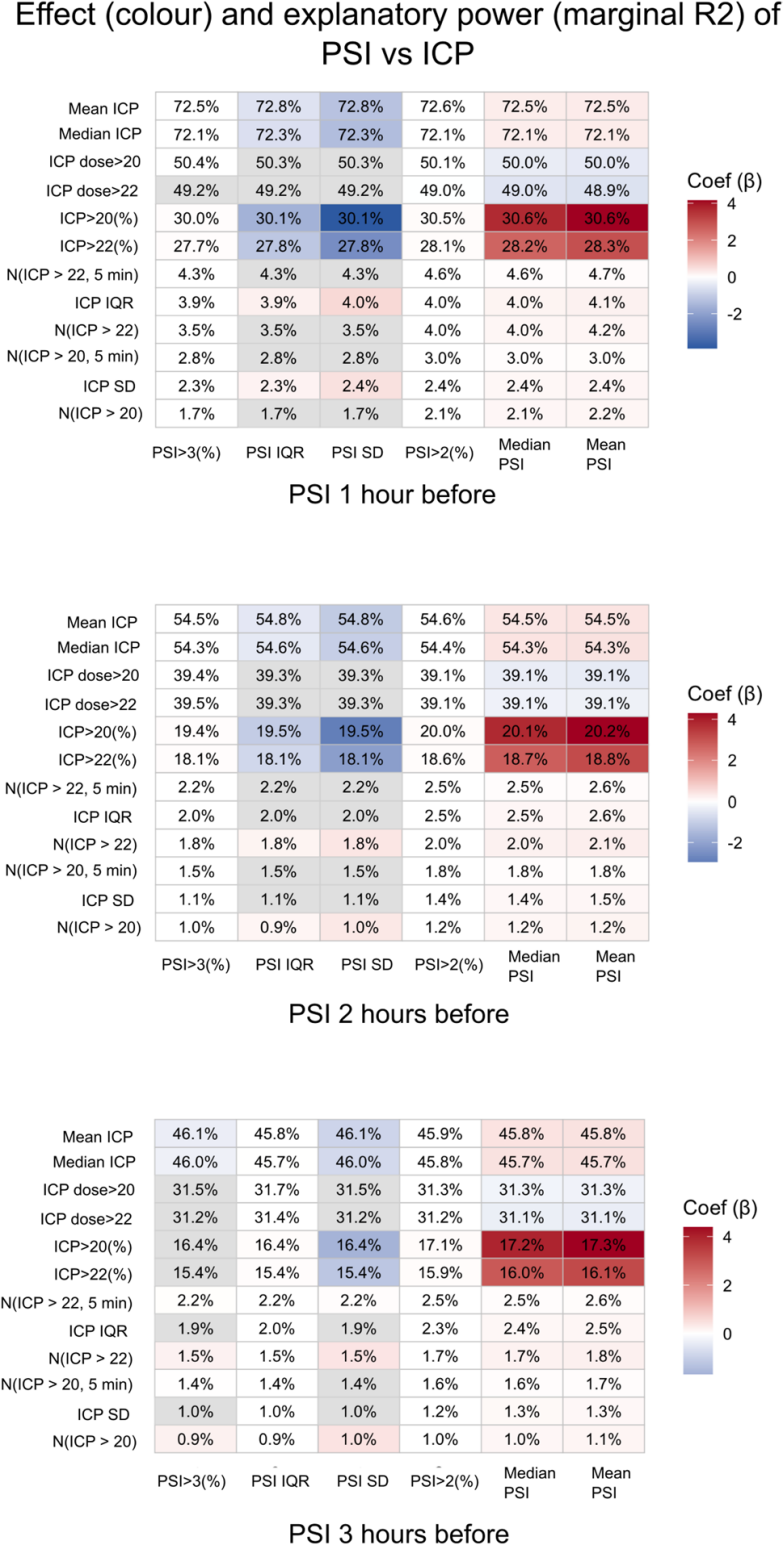
PSI as a predictor of future ICP crises. The figure shows the marginal R^2^ and β coefficients from the linear mixed-effects analyses examining associations between various PSI (predictor) and ICP (outcome) metrics. PSI metrics included mean/median values, %GMT above 2 and 3, respectively, and measures of variability (SD and IQR). ICP metrics encompassed mean/median ICP as well as several burden-based measures. ICP “dose” was defined as the area under the curve for values exceeding 20 and 22 mmHg, aligning with traditional and current guideline thresholds. We also assessed the %GMT above these thresholds, the number of ICP insults (any duration and those lasting ≥ 5 min), and ICP variability (IQR and SD). PSI was evaluated in three temporal windows preceding the ICP metrics: 1, 2, and 3 h. GMT = Good monitoring time. ICP = Intracranial pressure. IQR = Interquartile range. PSI = Pulse shape index. SD = Standard deviation

In Fig. 3, the associations between multiple PSI and ICP metrics across PSI lags of 1, 2, and 3 h are presented. These results were based on linear mixed-effects models using hourly averaged physiological data, with lagged PSI as the main predictor and mean ICP at the corresponding lag as a covariate. Higher PSI values were consistently associated with higher mean or median ICP, greater ICP dose > 20/22 mmHg, and a higher %GMT at ICP > 20/22 mmHg, with marginal R^2^ values of approximately 30% or higher when PSI was assessed 1 h before the ICP metric. While increased mean or median PSI showed a positive association with ICP burden, greater PSI variability (IQR or standard deviation [SD]) demonstrated an inverse association. As shown in the figure, the strength of the associations progressively weakened when PSI was evaluated 2 and 3 rather than 1 h before the ICP metric.

### PSI in relation to outcome

**Fig. 4:**
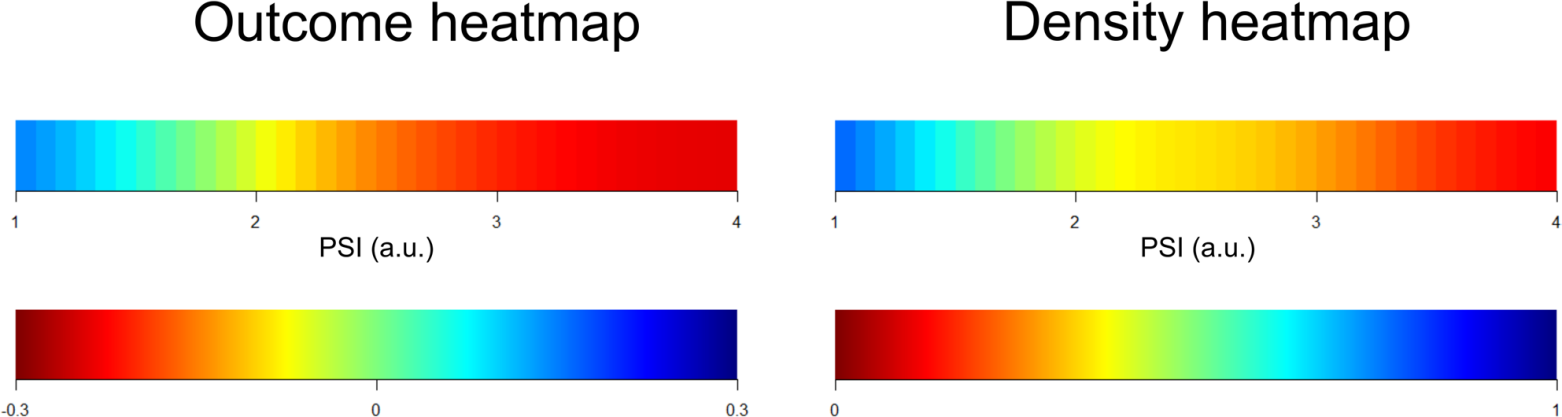
PSI – %GMT and intensity/duration in relation to outcome. Outcome heatmap – The outcome heatmap displays the colour-coded correlation coefficient (Spearman) between %GMT spent at each PSI interval and GOS (considered as an ordinal variable). Red indicates a negative association (i.e., higher %GMT linked to lower GOS/worse outcome), whereas blue indicates the opposite association. The scale is presented beneath the heatmap. Density heatmap – The density heatmap illustrates the distribution of PSI values across the dataset. Blue represents PSI values occurring with high frequency, while red indicates rare PSI values. The scale is presented beneath the heatmap. GMT = Good monitoring time. GOS = Glasgow Outcome Scale. PSI = Pulse shape index

**Fig. 5:**
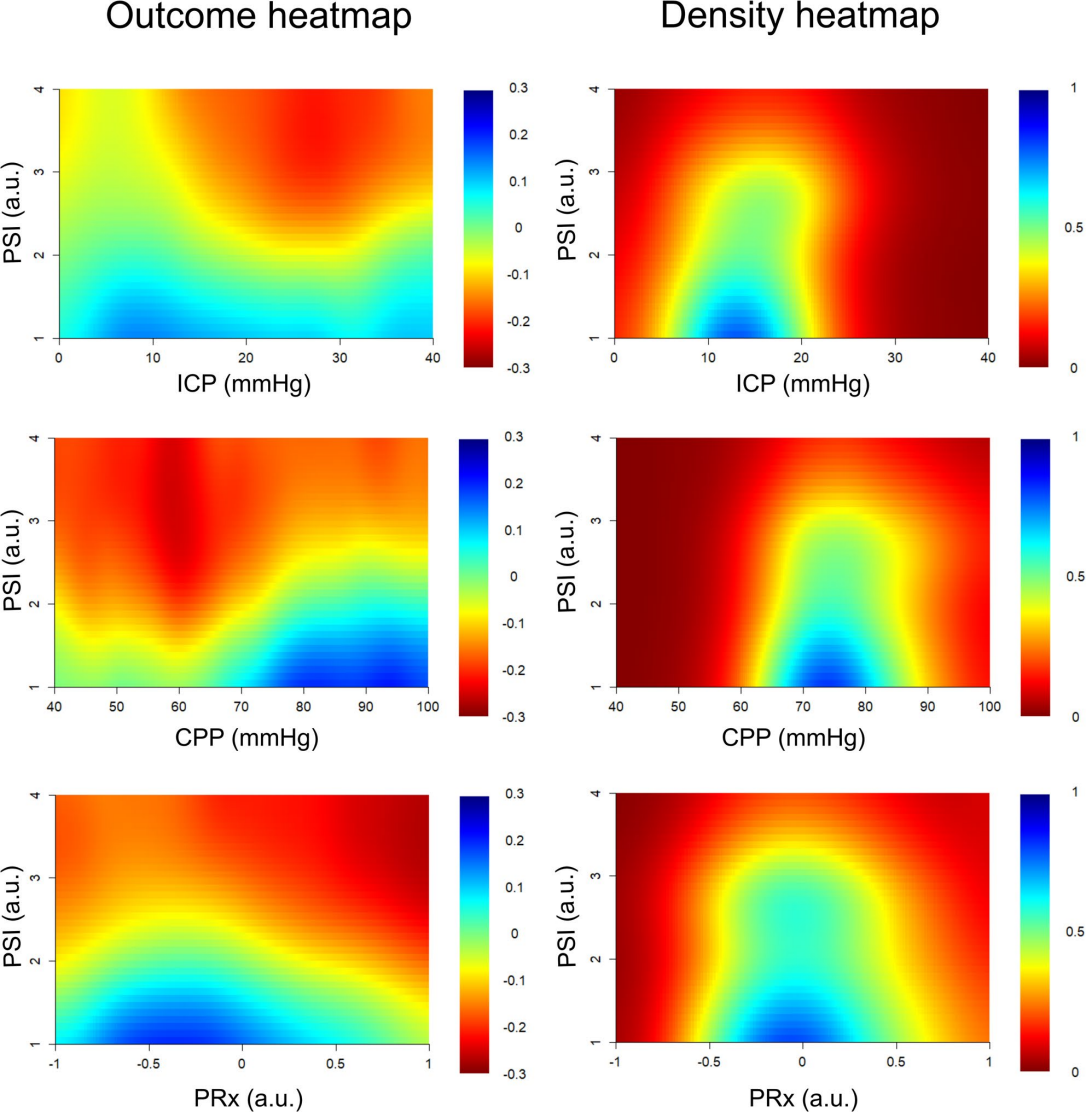
PSI in combination with intracranial pressure dynamics and cerebral autoregulation – relation to outcome. Outcome heatmaps – These figures show the correlation (Spearman) between the %GMT of PSI in combination with either ICP, CPP, or PRx within specific intervals in relation to outcome (GOS, considered as an ordinal variable). Red indicates a negative association (i.e., higher %GMT linked to lower GOS/worse outcome), whereas blue indicates the opposite association. The scale is presented on the left-hand side. Density heatmaps – These figures show the data distribution of PSI together with ICP, CPP, or PRx. The scale is presented on the right-hand side. CPP = Cerebral perfusion pressure. GMT = Good monitoring time. GOS = Glasgow Outcome Scale. ICP = Intracranial pressure. PRx = Pressure reactivity index. PSI = Pulse shape index

In univariable analysis, PSI was significantly lower in patients who survived as compared to those who died and in those with favourable vs. unfavourable outcome (Table 3). Furthermore, there was a transition towards worse outcome with higher PSI, particularly for values above 2, as demonstrated in Fig. 4. Most PSI values were confined to the range between 1 and 3, as indicated by the density heatmap. As illustrated in Fig. 5, high PSI in combination with high ICP, low CPP, and high PRx were particularly strongly associated with worse outcome, while a lower PSI in the latter scenarios mitigated that correlation. However, in multivariable analysis of mortality and favourable outcome (Table 4), PSI was not significantly associated with these dependent variables, both after adjustment for only clinical variables (age and GCS) and clinical together with physiological variables (ICP, PRx, and CPP). However, higher ICP and PRx were independently associated with increased mortality and a lower likelihood of favourable outcome, while lower CPP was independently associated with increased mortality only.

**Table 3:** PSI in relation to mortality and favourable outcome

Variables	Outcome groups		*P*-value
	Mortality	Survival	
PSI (a.u.), median (IQR)	2.0 (1.4–3.0)	1.6 (1.0–2.4)	***< 0.001***
PSI within threshold (%GMT)			
1.0–1.5, median (IQR)	19 (1–51)	46 (9–85)	***< 0.001***
1.5–2.0, median (IQR)	9 (2–21)	9 (3–18)	0.839
2.0–2.5, median (IQR)	11 (6–22)	7 (2–16)	***0.018***
2.5–3.0, median (IQR)	7 (3–15)	5 (1–18)	0.187
3.0–3.5, median (IQR)	12 (4–45)	3 (0–21)	***< 0.001***
3.5–4.0, median (IQR)	1 (0–10)	0 (0–1)	***< 0.001***

**Variables**	**Outcome groups**		**P-value**
	Unfavourable	Favourable	
PSI (a.u.), median (IQR)	2.0 (1.2–3.0)	1.4 (1.0–2.3)	***< 0.001***
PSI within threshold (%GMT)			
1.0–1.5, median (IQR)	30 (2–65)	55 (13–87)	***< 0.001***
1.5–2.0, median (IQR)	10 (3–19)	9 (3–18)	0.945
2.0–2.5, median (IQR)	10 (5–20)	6 (2–15)	***0.003***
2.5–3.0, median (IQR)	9 (2–17)	3 (0–17)	***0.003***
3.0–3.5, median (IQR)	9 (1–32)	1 (0–16)	***< 0.001***
3.5–4.0, median (IQR)	0 (0–4)	0 (0–0)	***< 0.001***

A.u. = arbitrary units. GMT = Good monitoring time. IQR = Interquartile range. PSI = Pulse shape index

**Table 4:** PSI in relation to mortality and favourable outcome – multivariable logistic outcome regression

**Variables**	**Mortality**			
	**Clinical variables + PSI**		**Clinical and physiological variables + PSI**	
	**OR (95% CI)**	**P-value**	**OR (95% CI)**	**P-value**
Age (years)	1.04 (1.02–1.06)	**< 0.001**	1.04 (1.02–1.07)	**< 0.001**
GCS (a.u.)	0.89 (0.80–0.98)	**0.02**	0.85 (0.76–0.95)	**0.005**
PSI (a.u.)	1.33 (0.86–2.05)	0.19	1.14 (0.68–1.90)	0.63
ICP (mmHg)	NA	NA	1.12 (1.05–1.21)	**0.002**
CPP (mmHg)	NA	NA	0.92 (0.86–0.97)	**0.007**
PRx (a.u.)	NA	NA	21.56 (4.29–121.54)	**< 0.001**

**Variables**	**Favourable outcome**			
	**Clinical variables + PSI**		**Clinical and physiological variables + PSI**	
	**OR (95% CI)**	**P-value**	**OR (95% CI)**	**P-value**
Age (years)	0.97 (0.95–0.98)	**< 0.001**	0.96 (0.94–0.98)	**< 0.001**
GCS (a.u.)	1.22 (1.13–1.32)	**< 0.001**	1.24 (1.14–1.35)	**< 0.001**
PSI (a.u.)	0.81 (0.56–1.16)	0.24	0.94 (0.64–1.40)	0.77
ICP (mmHg)	NA	NA	0.92 (0.87–0.97)	**0.002**
CPP (mmHg)	NA	NA	1.01 (0.97–1.05)	0.66
PRx (a.u.)	NA	NA	0.22 (0.06–0.84)	**0.03**

A.u. = arbitrary units. CI = Confidence interval. CPP = Cerebral perfusion pressure. GCS = Glasgow Coma Scale. ICP = Intracranial pressure. NA = Not applicable. OR = Odds ratio. PRx = Pressure reactivity index. PSI = Pulse hape index

## Discussion

In this study of 321 TBI patients, PSI was validated as a marker of intracranial compliance, showing associations with AmpICP, RAP, and ICP. Higher PSI also appeared useful for predicting future ICP elevations above 20/22 mmHg, on top of previous ICP mean values. Furthermore, higher PSI was linked to worse outcomes in univariable analyses, particularly above the threshold of 2. In two-dimensional heatmaps, elevated PSI appeared to narrow the ICP, PRx, and CPP ranges associated with more favourable outcomes. However, in multivariable outcome models adjusting for demographic factors, injury severity, and established NCC targets, PSI no longer retained independent prognostic value. Thus, it appears that outcome is more directly linked to established demographic (age) and NCC parameters than to PSI. Instead, the potential value of this metric more likely lies in its ability to predict future ICP insults, but this warrants further investigation. Moreover, further exploration of the physiological determinants that shape the ICP pulse waveform is essential to refine the classification of pathological waveform morphologies, which may ultimately strengthen both the definition and clinical utility of the PSI.

In this study, as expected, PSI deteriorated in parallel with other markers of impaired intracranial compliance. However, we noticed certain peculiar aspects in these analyses. At low AmpICP values (0–5 mmHg), PSI showed a volatile pattern with an early, prominent increase in PSI. One explanation is that AmpICP may be low both in states of very high and very low compliance, for example, during severely elevated ICP with cerebrovascular collapse, while PSI may better distinguish between these physiologically distinct states, potentially producing high PSI values even when AmpICP is low. Yet, excluding episodes with extreme ICP did not alter the GAM findings. Another explanation is that both the ICP pulse amplitude and PSI are closely linked to age [15], with the volatile pattern at low AmpICP being most evident in older patients in our study. Vascular ageing and related disease may alter the transmission of cardiac stroke volume to the ICP pulse waveform in ways that primarily reflect arterial and parenchymal properties rather than intracranial compliance itself [10], potentially producing apparently pathological pulse wave characteristics despite preserved compliance. At present, however, the physiology underlying the ICP pulse waveform remains incompletely understood and warrants further clarification [36]. It also remains possible that waveform classification becomes less reliable at very low amplitudes, thereby reducing the discriminatory performance of PSI, and that GAMs, being sensitive to noise, further amplified this apparent volatility. Beyond this low-amplitude range, however, PSI increased linearly with higher AmpICP, as expected.

PSI also worsened modestly with increasing RAP, particularly between + 0.5 and + 1.0, consistent with the range in which intracranial compliance is thought to decline. Likewise, PSI remained relatively stable at ICP values of 0–15 mmHg but increased steadily at higher ICP levels, again corresponding to when intracranial compliance is expected to become exhausted. PSI additionally increased with higher ABP, consistent with previous work [15], as elevated ABP may reflect increased cardiac stroke volume, stress, or vascular disease [37], all of which can influence transmission of pulsatile pressure to the intracranial compartment [15, 36]. Altogether, PSI appears to reflect intracranial compliance in a manner broadly consistent with established metrics, although all such measures are influenced by multiple physiological factors and therefore capture overlapping but not identical information. In particular, age- and vascular-related effects on PSI merit further investigation.

Previously, PSI has proven to be useful in predicting the extent of radiological mass effect in TBI patients [14, 19], and may even outperform mean ICP in this regard [19]. In this study, we found that higher PSI values had relatively strong explanatory value in predicting the risk of developing ICP insults (values > 20/22 mmHg). In this association, PSI captured information from the ICP waveform that was not reflected in mean ICP alone, indicating that the two measures provide complementary physiological insights [14]. This association was most pronounced when PSI was evaluated during the hour immediately preceding the crisis, whereas longer temporal lags were associated with a progressive reduction in predictive ability, an expected finding given the rapid fluctuations that characterise ICP dynamics. These observations support the notion that ICP pulse waveform characteristics, including PSI, may add important information and should be included in future AI-based methods for forecasting ICP insults. We also observed that greater PSI variability (IQR or SD) corresponded to a lower burden of ICP elevation. This finding is somewhat unexpected, as increased PSI variability could be hypothesized to reflect a system that remains on the steeper portion of the intracranial pressure–volume curve, where small changes in intracranial volume are expected to produce larger changes in ICP and, potentially, resulting in intracranial hypertension [7, 14, 25]. However, it is also possible that the morphology of the ICP pulse waveform begins to change, or becomes more variable, *before* the system reaches the steep portion of the curve, whereas once on the steep segment the waveform may instead become more persistently abnormal or fixed in its disturbed configuration. Additionally, greater physiological variability is often considered a favourable feature [38, 39], and higher PSI variability may indicate a healthier intracranial system. It may also reflect that the patient remains more responsive to therapeutic optimizations. In such circumstances, PSI may fluctuate more widely because the brain’s compensatory mechanisms are still active rather than exhausted. In summary, these observations suggest that both the absolute level and the dynamic behaviour of PSI provide clinically relevant information for predicting ICP problems.

In univariate analysis, as anticipated, higher PSI was associated with increased mortality and a lower probability of favourable outcome. While PSI values clustered around the range of normal waveform morphology (1.0–1.5), the transition to worse outcomes appeared to occur already at values suggestive of early pathological changes (above 2), while extreme cases with PSI around 4 remained relatively rare. The two-variable heatmaps further illustrated that combinations of high ICP and high PSI were particularly unfavourable, whereas low values of both were associated with more favourable outcomes. Notably, low PSI appeared to mitigate the adverse effects of high ICP on outcome, whereas elevated PSI was associated with a shift towards poorer outcomes in the context of low ICP. This may suggest that impaired intracranial compliance, reflected by elevated PSI, could in itself be detrimental, independent of ICP level. However, in multivariable regression models of both mortality and favourable outcome, PSI lost its independent prognostic value after adjustment for clinical variables (age and GCS) and NCC targets (ICP, PRx, and CPP). This attenuation may reflect that elevated PSI is correlated with both older age and higher ICP [14, 15], suggesting that its apparent association with worse outcomes is primarily driven by reduced functional reserve in older patients and the pathological effects of sustained intracranial hypertension. It is important to underscore that these findings pertain to retrospective analyses, where ICP data are already available. In contrast, as outlined above, PSI may still be useful in a prospective perspective, as an alert of an increased risk of mass lesions and future ICP problems. Thus, while ICP, PRx, and CPP remain the primary NCC targets and are most strongly associated with outcome, PSI may still hold clinical value as an early warning indicator, particularly for anticipating future ICP problems.

### Methodological considerations

This study had several notable strengths. While previous evaluations of PSI have primarily been conducted within the CENTER-TBI cohort, a valuable but relatively limited multi-centre dataset comprising approximately 150 patients, our analysis was based on a considerably larger, although single-centre, TBI cohort. We validated prior findings in physiological domains and extended the analysis through advanced statistical and visualization methods. It should be noted that a small subset of patients (*n* = 10, 3% of our study population) in the present cohort also was included in previous CENTER-TBI studies (from the Cambridge site) on PSI [14, 15, 19], although this overlap is limited and unlikely to have materially influenced the overall findings. Some limitations should also be acknowledged. Legal restrictions precluded access to more detailed clinical data, such as radiological assessments of mass lesions. Additionally, data on DC were incomplete for a substantial number of patients, necessitating their exclusion from parts of the analysis. Consequently, the study population represents a selected cohort, restricted to patients with available outcome data and those with known DC status, including analyses limited to patients in whom DC was not performed. Also, as this was a single-centre study from a highly specialised NCC unit, the findings may not be directly generalisable to centres with different patient populations, monitoring practices, or treatment protocols. In addition, as with all observational and model-based analyses, and residual confounding or model misspecification cannot be entirely excluded. Furthermore, the use of time-averaged data may have reduced sensitivity to short-term physiological fluctuations, although it enabled more robust assessment of temporal trends. A further limitation concerns the automated nature of waveform classification. Although the neural network classifier used in this study has been previously validated [20], residual artifacts and occasional misclassification cannot be entirely excluded and may introduce bias into PSI calculations. Additionally, the 4-class waveform categorization, while balancing robustness with interpretability, may not fully capture the complexity of all observed morphologies. Validation against expert annotation represents an important direction for future work; however, such a study requires careful design to account for inter-observer variability inherent in the manual review of physiological signals. Lastly, this study included patients treated over a 20-year period [22], during which only minor variations occurred in management protocols. The most notable change, in the light of this analysis, was the adjustment of the ABP reference level, which may have influenced the GAM analyses involving ABP in relation to PSI to some extent.

## Conclusions

In this study, PSI was validated as a marker of impaired intracranial compliance, showing clear associations with ICP, AmpICP, and RAP. Higher PSI also appeared useful for predicting future ICP insults and was linked to worse outcomes in univariable analyses, particularly above a threshold of 2. However, in multivariable outcome models adjusting for demographic factors and established NCC targets, PSI no longer retained independent prognostic value. Thus, retrospectively, outcome appears more directly influenced by age and ICP than by PSI which is not surprising given the underlying physiological meaning of the variables. However, the potential value of PSI likely lies in its prospective ability to signal deteriorating intracranial compliance and predict impending ICP insults, an area that warrants further investigation. In addition, it remains of essence to improve our understanding of the features that characterize the ICP pulse waveform morphology in order to refine PSI definitions and enhance its clinical utility.

## Supplementary Information

Supplementary Material 1

## Data Availability

Data are available upon reasonable request.

## Abbreviations

AI: Artificial intelligence
AmpICP: Peak-to-peak ICP pulse amplitude
A.u.: Arbitrary unit
ABP: Arterial blood pressure
CA: Cerebral autoregulation
CBF: Cerebral blood flow
CI: Confidence interval
CPP: Cerebral perfusion pressure
DC: Decompressive craniectomy
GAM: Generalised additive models
GCS: Glasgow Coma Scale
GMT: Good monitoring time
GOS: Glasgow Outcome Scale
ICP: Intracranial pressure
IQR: Interquartile range
NCC: Neurocritical care
OR: Odds ratio
PRx: Pressure reactivity index
PSI: Pulse shape index
RAP: The correlation (r) between amplitude and ICP
SD: Standard deviation
TBI: Traumatic brain injury
