# Supplementary material for: Intracranial compliance monitoring using pulse shape index in traumatic brain injury: relation to cerebral physiology and clinical outcome
Source: Crit Care. 2026 May 9;30:247. doi: 10.1186/s13054-026-06071-0 (PMC13159340; doi:10.1186/s13054-026-06071-0)
Supplement: Supplementary file 1 — Supplementary Material 1 [file 13054_2026_6071_MOESM1_ESM.pdf]

Additional file 1. PSI in relation to AmpICP and RAP after exclusion of extreme ICP values

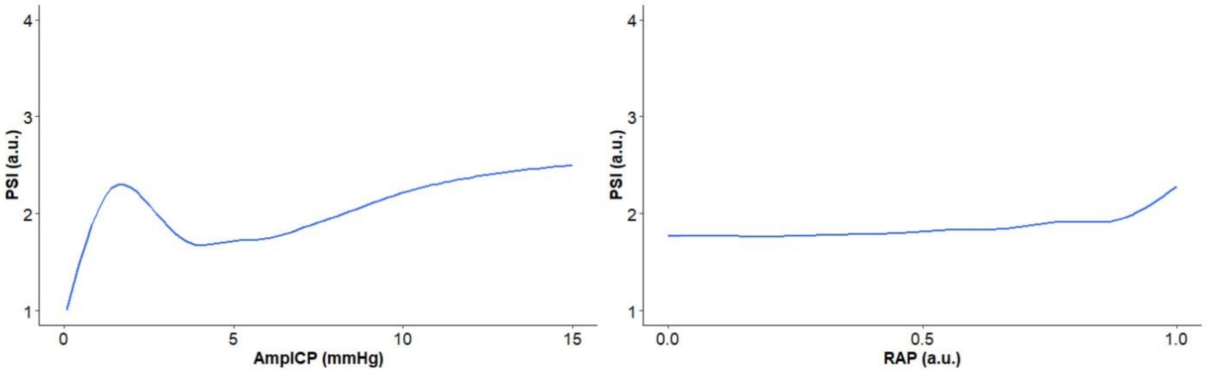

These sensitivity analyses illustrate the potentially non-linear relationships between PSI and AmpICP and RAP, as modelled using GAMs. Each analysis was restricted to physiologically relevant ranges: PSI 1-4, AmpICP 0-15 mmHg, and RAP 0.0-+1.0. To reduce confounding from extreme intracranial hypertension, only data points with concurrent ICP below 40 mmHg were included.

AmpICP = peak-to-peak ICP pulse amplitude. A.u. = Arbitrary unit. GAM = Generalised additive models. ICP = Intracranial pressure. PSI = Pulse shape index. RAP = the correlation (r) between ICP amplitude and ICP.
